# Hemodynamics of Prefrontal Cortex in Ornithine Transcarbamylase Deficiency: A Twin Case Study

**DOI:** 10.3389/fneur.2020.00809

**Published:** 2020-08-14

**Authors:** Afrouz A. Anderson, Andrea Gropman, Cynthia Le Mons, Constantine A. Stratakis, Amir H. Gandjbakhche

**Affiliations:** ^1^National Institutes of Health (NIH), National Institute of Child Health and Human Development, Bethesda, MD, United States; ^2^Children's National Medical Center, Division of Neurogenetics and Neurodevelopmental Pediatrics, Washington, DC, United States; ^3^National Urea Cycle Disorders Foundation, Pasadena, CA, United States

**Keywords:** Ornithine transcarbamylase deficiency, functional near infrared spectroscopy, urea cycle disorders, cerebral autoregulation, fNIRS, functional connectivity, wavelet coherence

## Abstract

Ornithine transcarbamylase deficiency (OTCD) is the most common form of urea cycle disorder characterized by the presence of hyperammonemia (HA). In patients with OTCD, HA is known to cause impairments in domains of executive function and working memory. Monitoring OTCD progression and investigating neurocognitive biomarkers can, therefore, become critical in understanding the underlying brain function in a population with OTCD. We used functional near infrared spectroscopy (fNIRS) to examine the hemodynamics of prefrontal cortex (PFC) in a fraternal twin with and without OTCD. fNIRS is a non-invasive and wearable optical technology that can be used to assess cortical hemodynamics in a realistic clinical setting. We quantified the hemodynamic variations in total-hemoglobin as assessed by fNIRS while subjects performed the *N*-back working memory (WM) task. Our preliminary results showed that the sibling with OTCD had higher variation in a very low frequency band (<0.03 Hz, related to mechanism of cerebral autoregulation) compared to the control sibling. The difference between these variations was not as prominent in the higher frequency band, indicating the possible role of impaired autoregulation and cognitive function due to presence of HA. We further examined the functional connectivity in PFC, where the OTCD sibling showed lower interhemispheric functional connectivity as the task load increased. Our pilot results are the first to show the utility of fNIRS in monitoring OTCD cortical hemodynamics, indicating the possibility of inefficient neurocognitive function. This study provides a novel insight into the monitoring of OTCD focusing on the contribution of physiological process and neurocognitive function in this population.

## Introduction

The urea cycle disorders are a group of rare genetic disorders caused by a deficiency of the enzymes or transport proteins that remove ammonia from the body. Ornithine transcarbamylase deficiency (OTCD) is an X-linked inborn error of metabolism and the most common of the urea cycle disorders (1). Deficient protein metabolism in OTCD results in hyperammonemia (HA) that, in turn, disrupts the neurocognitive function in domains of working memory and executive function in several brain regions, including prefrontal cortex (PFC) (2–4). Investigation of neurocognitive function in working memory domain can become critical to understand the underlying effects of hyperammonemia in patients with OTCD.

In this study, we use functional near infrared spectroscopy (fNIRS) as a non-invasive optical methodology to investigate the neurocognitive function based on hemodynamics of the cortical region. fNIRS uses the light in the near infrared region to measure the changes in oxy-hemoglobin and deoxy-hemoglobin as two biomarkers of brain function (5–7). Given that using functional magnetic resonance imaging (fMRI) in examining the neurocognitive function can be difficult in a pediatric group, especially those with cognitive disorders, using wearable, and non-invasive optical technology allows the assessment of useful hemodynamic biomarkers in a realistic and clinical setting without restricting the participants' motion.

Underlying physiological mechanisms can result in hemodynamic oscillations at different frequency ranges (8, 9). Cerebral autoregulation (CA) is the critical cerebrovascular mechanism to ensure stable and regulated cerebral hemodynamics in the brain. CA maintains the cerebral blood flow by means of vasomotion and has been shown to be related to brain function in typical development (10–13) as well as those with developmental delays and poor neurocognitive outcomes (14–16). Abnormalities in cerebral autoregulation have been reported in several conditions, such as dementia, mild traumatic brain injury, and stroke. Due to hyperammonemia in OTCD and its potential effects on cerebrovascular function (17, 18), exploring the frequency range of CA can be useful to understand the neurocognitive function and development in this population. It has been shown that hemodynamic oscillations in very low frequency range, such as 0.03 Hz, can be related to the physiological range of cerebrovascular mechanism and autoregulation (19, 20).

Investigating brain functional connectivity (FC) can further provide a possible biomarker of brain development (21). FC defines the functional relationship between distinct brain regions and can be analyzed in terms of correlation between the temporal changes in hemodynamic response. The concept of FC has been used in studying developmental disorders such as autism spectrum disorder (ASD) and attention deficit hyperactivity disorder (ADHD) (22, 23) as well as other clinical manifestation, such as acute ischemic stroke (24). Reduced functional connectivity during the resting state in medical PFC and other regions, such as the amygdala, in subjects with autism has also been shown (25). In mild traumatic brain injury, FC has been shown as a useful tool to provide objective biomarkers of cognitive impairments (26). Decreased interhemispheric functional connectivity in PFC has also been found in several other disorders, such as stroke, affective disorder, and gaming disorder (27–29).

To explore FC and cortical hemodynamics related to cerebral autoregulation, we use fNIRS as a non-invasive and wearable modality (13, 30). fNIRS allows assessment of changes in oxy-hemoglobin (HbO) and deoxy-hemoglobin (Hb) as the light in the near infrared region (700–900 nm) penetrates through cortical regions. The capability of fNIRS assessing cortical hemodynamics and FC makes it a promising tool for potential monitoring and detection of possible early differences in neurodevelopmental and neurocognitive disorders.

In this study, we examine the hemodynamic features as well as the functional connectivity in prefrontal cortex in a set of twins, one with and one without OTCD. Since OTCD is considered a rare disease (1 in 80,000) and, therefore, twin studies in this population are extremely rare, this case study would provide the opportunity to examine the confounding cognitive biomarkers while reducing the factors that could affect the cognitive function, such as variations in age and environment.

Given that hyperammonemia in OTCD can affect the hemodynamics and underlying neurocognitive function (31, 32), we hypothesize that such effects can be manifested in the hemodynamic signal and its features as measure by fNIRS. We hypothesize that we will observe differences in hemodynamic oscillations and functional connectivity in PFC during performance of working memory task due to effect of hyperammonemia on global cognitive function in the sibling with OTCD. We further examine hemodynamic oscillations at different frequency bands related to mechanism such as cerebral autoregulation to further investigate the role of such features in OTCD.

## Materials and Methods

### Participants

Participants in this study were two 11-year-old fraternal twins, a male and a female. The female sibling was diagnosed as a symptomatic OTCD carrier at young age, and the male sibling participated as control and did not have any OTCD-related symptoms or carry a gene mutation. The study is reviewed and approved by The Children's National Medical Center Institutional Review Board. Written informed consent to participate in this study was provided by the participants' legal guardian.

The twins were born after an uncomplicated pregnancy and delivery at 32 weeks due to premature rupture of the membrane in the male twin sack. They were born by caesarian section and did not require oxygen or ventilation in the delivery room. They were discharged at term equivalent age with no medical complications. The birth weight was 2041.17 g for the female and 2579.81 g for the male. The female experienced coma at 3 days of age, which retrospectively was noted to be due to possible protein intolerance. No workup was performed until she was diagnosed at 6 months of age due to continuous vomiting, spiting, fussiness, and delayed growth at the time of diagnosis, which previously had been attributed to viral infection. However, by the age of 4 years, she was caught up on the growth chart. The twins were in a NICU follow-up clinic for the first year of life with no concerns.

Her highest ammonia recorded was 420 in 2011 although she had >20 hospitalizations from 2008–2016 many with hyperammonemia (<400) associated with intercurrent infections. The liver function, AST and ALT were at 2,000 U/L. She was protein restricted since diagnosis on 10 g/day. Currently she is managed with glycerol phenylbutyrate (Ravicti) since 2011 as well as a protein restricted diet (15 g/day) citrulline and prophree formula and essential amino acids.

There is no history of hemodialysis, seizures, head trauma, or other neurological concerns. There was no MRI finding as it was not a standard procedure in evaluation of UCD patients. Developmental milestones, such as walking, were delayed and started at ~13–14 months old compared to the male sibling ~8 months. Both children are right-handed. Neither have had formal IQ testing. Neither subjects had educational difficulties and are performing well at the appropriate grade level at school. There were no clinical or developmental concerns for the male sibling. The twins' medical history and hospitalization are shown in Table 1.

**Table 1:** Participants' medical history.

	**Age (years)**	**Gender**	**Handedness**	**Time of diagnosis (mo.)**	**Hospitalization**	**Highest plasma ammonia level**	**Estimated first walk (mo.)**	**Birth weight (g)**	**Weight at 6 months (g)**
OTCD sibling	11	Female	Right	6	>20	420	13–14	2041.17	4535.9
Control sibling	11	Male	Right	-	-	-	8	2579.81	6803.89

Familial mutation testing revealed heterozygous missense mutation c.482A>G (p.N161S) in the affected female twin. The mother, who remains asymptomatic, does not have any protein-restriction diet or intolerance or any documented hyperammonemia. She did not have excessive nausea or vomiting during the pregnancy. Ammonia level in the mother was not obtained since there was no symptoms reported. The maternal grandmother was tested and does not harbor the mutation.

### Task

**Figure 1:**
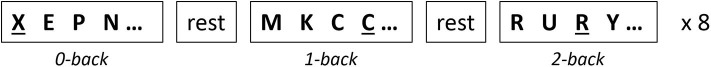
Illustration of the N-back working memory task. In the 0-back condition, the target letter is “X” (indicated with underline). In the 1- and 2-back conditions, the target letter is the same as the letter from 1- or 2-steps before in a given sequence, respectively. Each condition was repeated eight times with the rest period in between conditions.

The *N*-back working memory (WM) task was used to induce activation in prefrontal cortex (PFC). This task is based on the continuous variable design, where the level of task difficulty increases from easy to hard (0-back to 2-back). This design allows us to examine the brain activation and behavioral response at different levels of cognitive demands. Participants performed three level of the WM task: 0-, 1-, and 2-back. In all conditions, a sequence of letters (stimuli) appears in the center of a screen for 800 ms with a 1,200-ms interstimulus interval. In the 0-back condition, subjects were instructed to press a handheld keyboard whenever they observed the letter “X.” For the 1- and 2-back conditions, subjects clicked whenever the target letter was same as the 1 and 2 steps before, respectively. Each condition was repeated eight times (total of 24 blocks) with a duration of 20 s and with a rest period of 10 s between each condition. The details of task design are illustrated in Figure 1. The *N*-back test was designed using E-Prime software and was displayed on a 15-inch monitor. Subjects were asked to press the designated button when they observed the target letters. Behavioral measures, such as reaction time and accuracy, were collected through the E-Prime software.

### Data Acquisition and Analysis

**Figure 2:**
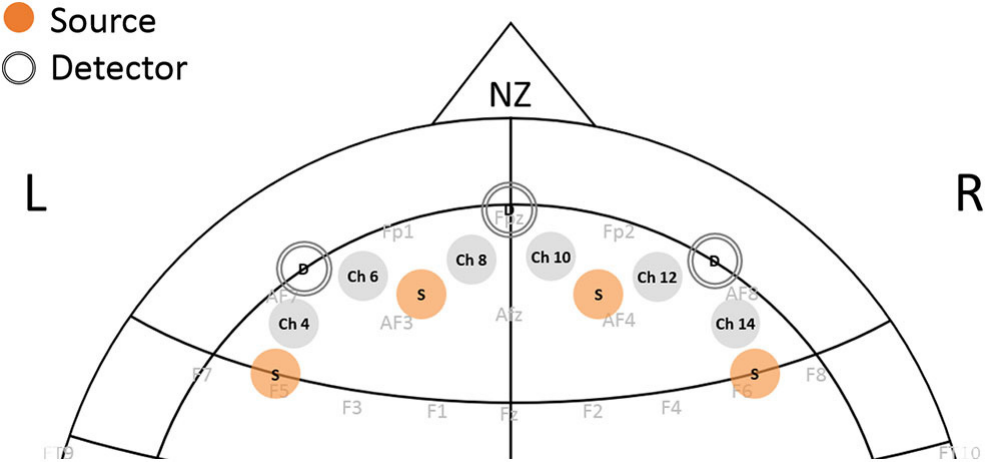
Position of fNIRS sensor on the forehead. Each channel represents the source-detector pair measuring the changes in oxy-hemoglobin and deoxy-hemoglobin from prefrontal cortex regions.

Before starting the task, subjects were asked to sit comfortably and at the distance of ~2 feet from the monitor. An fNIRS sensor consisting of four sources and 10 detectors with a total of 16 source–detector pairs (channels) was positioned on the subjects' forehead (fNIRS Devices, LLC) with the middle of sensor matching on the fPz location based on the 10–20 system. Detectors to the left and the right were located on the AF7 and AF8 locations, respectively, while sources from left and right were covering the regions of F5, AF3, AF4, and F6, respectively (Figure 2). The raw intensity data were recorded using Cobi Studio software (33). Changes in Hb and HbO were calculated using the modified Beer-Lambert law with values of differential path length factor calculated based on the subjects' age (34). The 10-s rest period prior to the start of the task was used as a baseline to calculate the changes in HbO and Hb signals. In a continuous wave system, we obtain the changes in oxy- and deoxy-hemoglobin signal with respect to the baseline prior to the start of the task for each participant. Therefore, constant effects in each participant, such that those by skin and scalp, are reduced, and task-related effects on skin blood flow have been shown to be negligible (35). We further use a source–detector separation of 2.5 cm that allows for differentiation of signals coming from the cerebrum vs. skin. Subjects were instructed to relax during this rest period as much as possible. Median filtering was applied to the HbO and Hb signals to remove sharp spikes in the data (6). The blocks from each condition were appended temporally. Channels 4, 6, 8, 10, 12, and 14, which covered the bilateral prefrontal region, were included in analysis (Figure 2). To account for changes in both oxy-hemoglobin (HbO) and deoxy-hemoglobin (Hb), the total hemoglobin (HbT, HbT = HbO + Hb) was used. From here, the data was filtered in several frequency bands using a bandpass Butterworth filter: very low frequency band 1 (VLF1: 0.001–0.03 Hz), very low frequency band 2 (VLF2: 0.001–0.1), and low frequency (LF: 0.07–0.1). The bandpass filter further removed the effect of heart-rate and respiration frequencies. Task length was ~16 min long, allowing capturing hemodynamic activity at the low frequency. We applied the concept of complex signal analysis using Hilbert transform to calculate instantaneous amplitude of the HbT signal (13, 36). We then examined hemodynamic variation (HV), as a hemodynamic feature by calculating the coefficient of variation of the signal that results in unitless index such that $HV=\frac{\sigma \left(inst_{signal}\right)}{\mu \left(inst_{signal}\right)}$, where σ and μ represent standard deviation and mean of the given signal. HV characterizes the instantaneous oscillations in total hemoglobin in a frequency band of choice. The utility of using this coefficient has been shown previously (13, 15, 16).

For the functional connectivity analysis, wavelet coherence analysis as a measure of correlation between two signals was used to find the strength of the correlation between time series HbT data between channels. Wavelet coherence analysis provides the correlation between two signals in both time and frequency domains and has been described previously (37, 38). The coherence values were calculated using the Matlab Wavelet Toolbox between each perspective channel and were averaged over the given temporal window.

The values of phase coherence coefficient are between 0 and 1. When the two signals are not correlated, the phase coherence coefficient approaches 0. The higher correlation values would indicate the stronger temporal correlation between the given two channels with values of 1 indicating the highest correlation. The correlation matrix based on these coherence values for each set of tasks were created. After finding the correlation matrix, thresholding was applied to distinguish the channel with the correlations above 0.6.

## Results

### Behavioral

Table 2 shows the *N*-back task performance for the OTCD and control siblings. As expected, the reaction time was slower for the OTCD sibling. The percentage correct response was also lower compared to the control sibling. Both siblings had a lower number of correct responses during performance of the 2-back task. Between subjects' responses were similar during the less demanding 0-back task, and the differences were more prominent during more challenging conditions, such as 1- and 2-back.

**Table 2:** N-Back task performance and reaction time in OTCD and control siblings.

	**Age (years)**	**Gender**	**% correct response 0-back**	**% correct response 1-back**	**% correct response 2-Back**	**Reaction time (ms) 0-back**	**Reaction time (ms) 1-back**	**Reaction time (ms) 2-back**
OTCD sibling	11	Female	91.67	40.91	37.50	707.50	1120.78	1079.44
Control sibling	11	Male	95.83	81.82	58.33	560.65	630.56	527.64

### Hemodynamic Variation (HV)

**Figure 3:**
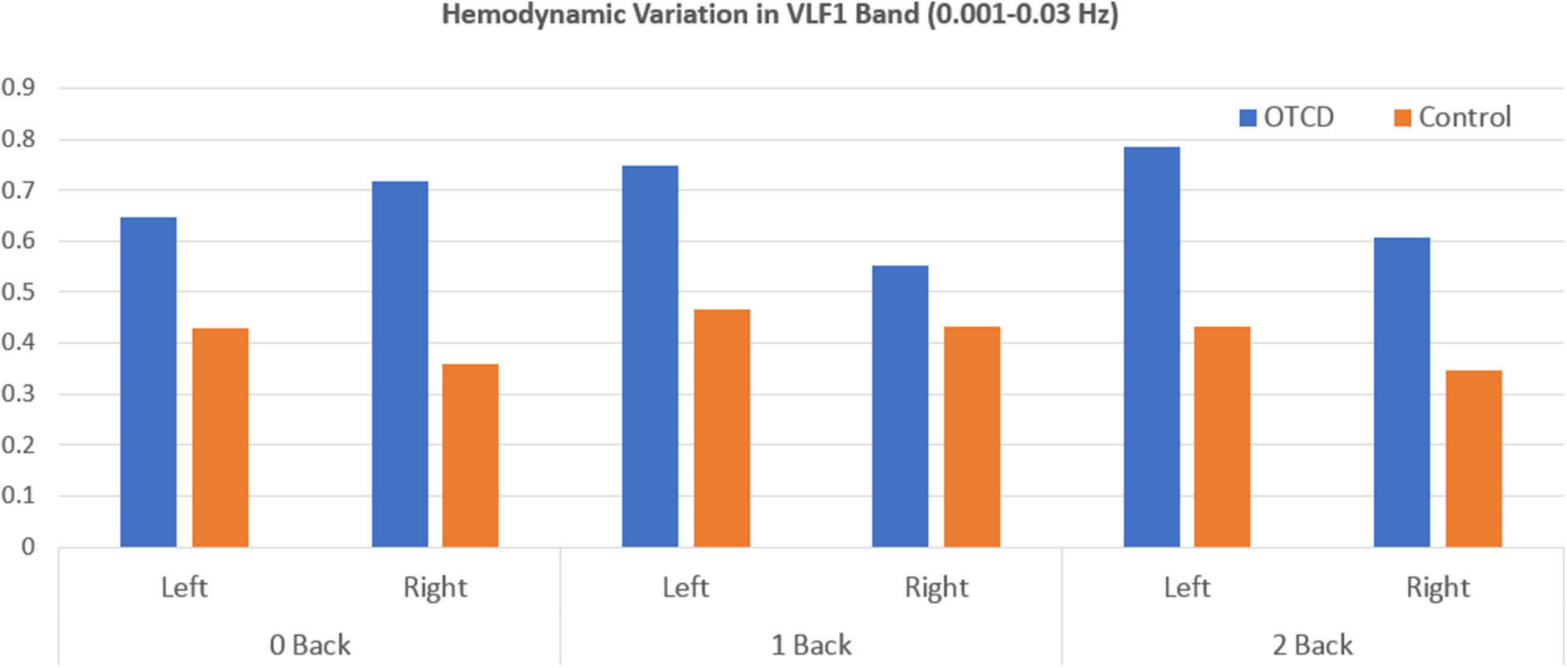
Hemodynamic variation based on the coefficient of variation of total hemoglobin in the very low frequency range (0.001–0.03 Hz) during performance of WM task from left and right prefrontal cortex region.

Hemodynamic variabilty values based on the coefficient of variation in the HbT signal from each condition were calculated. Figure 3 shows the HV values for the VLF1 for all task conditions. Overall, the sibling with OTCD showed a higher HV index in both left and right prefrontal cortex. The difference between the indices was more prominent in the left prefrontal cortex and during tasks with higher cognitive working memory (WM) demand. Although, during the 0-back condition, the right PFC showed a higher difference between the HV values, during more challenging tasks, such difference became more apparent in left PFC.

**Figure 4:**
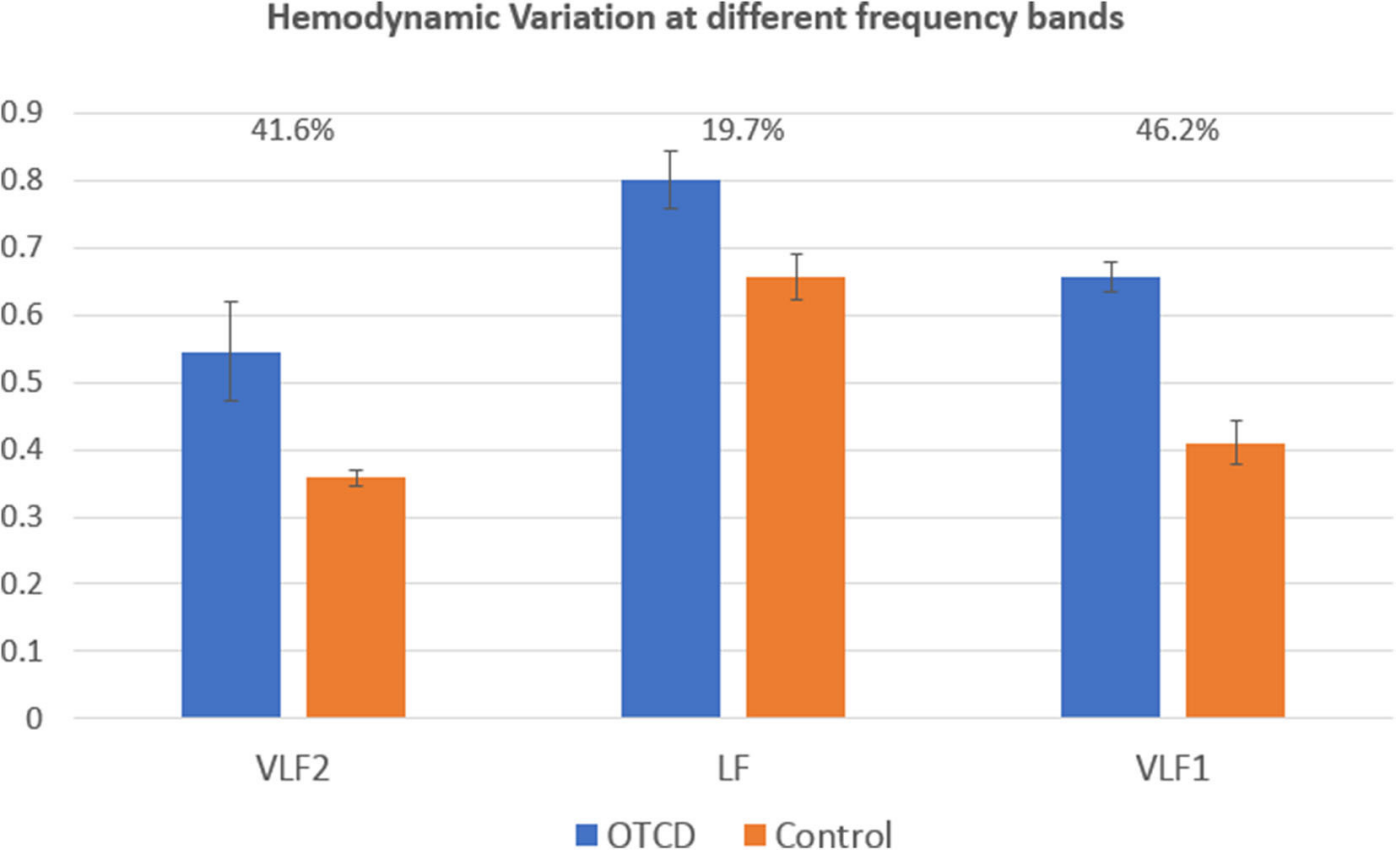
Comparison of hemodynamic variation at different frequency bands. The VLF1 frequency band (0.001–0.03) shows the highest difference between hemodynamic variation between the sibling with and without OTCD.

Figure 4 shows the HV values for the control and OTCD twins at different frequency bands of VLF1 (0.001–0.03 Hz), VLF2 (0.001–0.1), and LF (0.07–0.1). For simplicity, the HV has been averaged across all conditions. These results show that, for a frequency band of 0.07–0.1 Hz (LF), the hemodynamic variation does not show a distinct difference between control and OTCD subject (difference of 19.7%). On the other hand, the largest difference was detected for the VLF of 0.001–0.03 Hz, where the observed difference was 46.2%.

**Figure 5:**
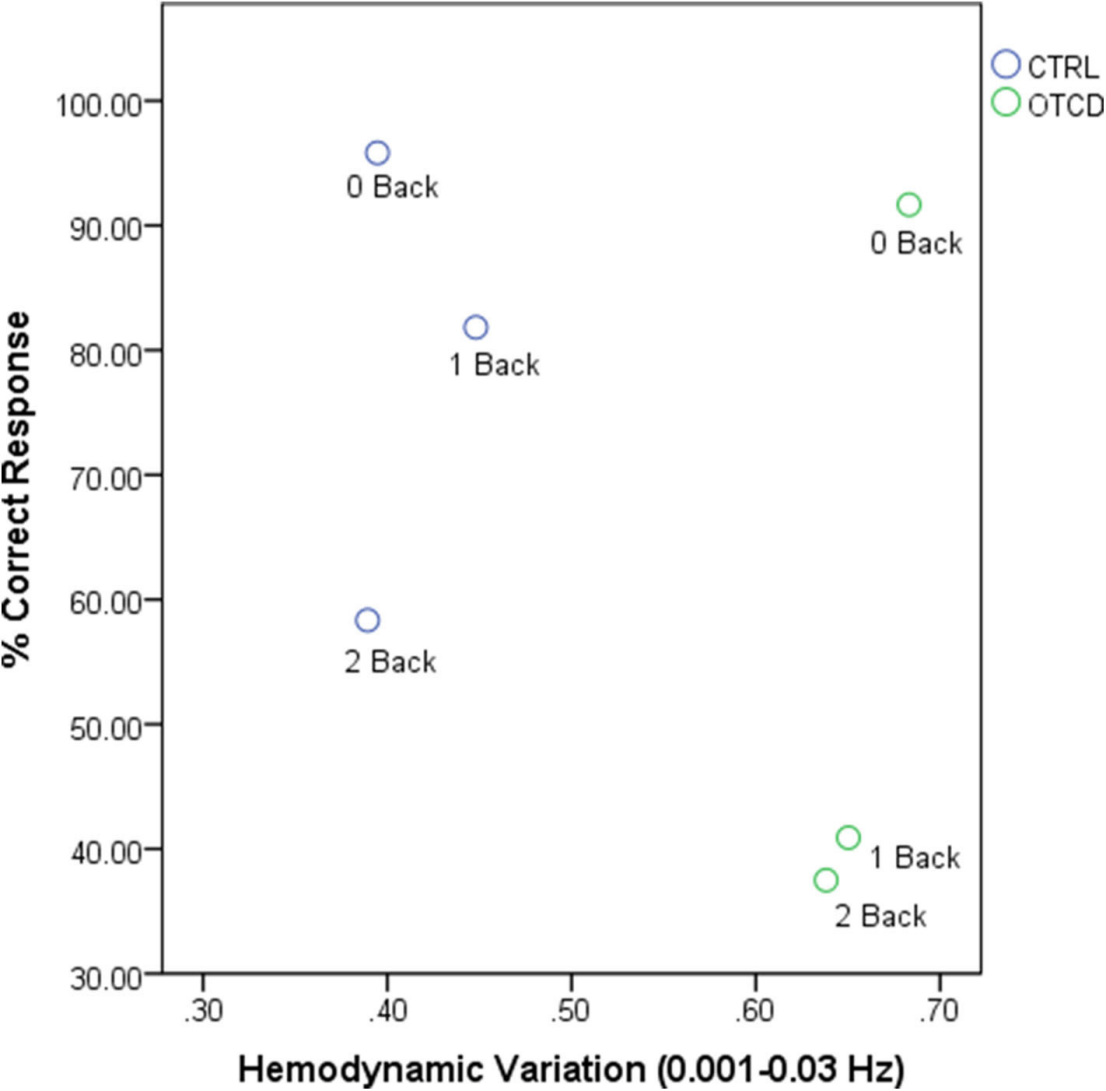
Relationship between hemodynamic variations and task performance for OTCD and control sibling.

The relationship between hemodynamic variations and task performance is shown in Figure 5.

### Functional Connectivity

**Figure 6:**
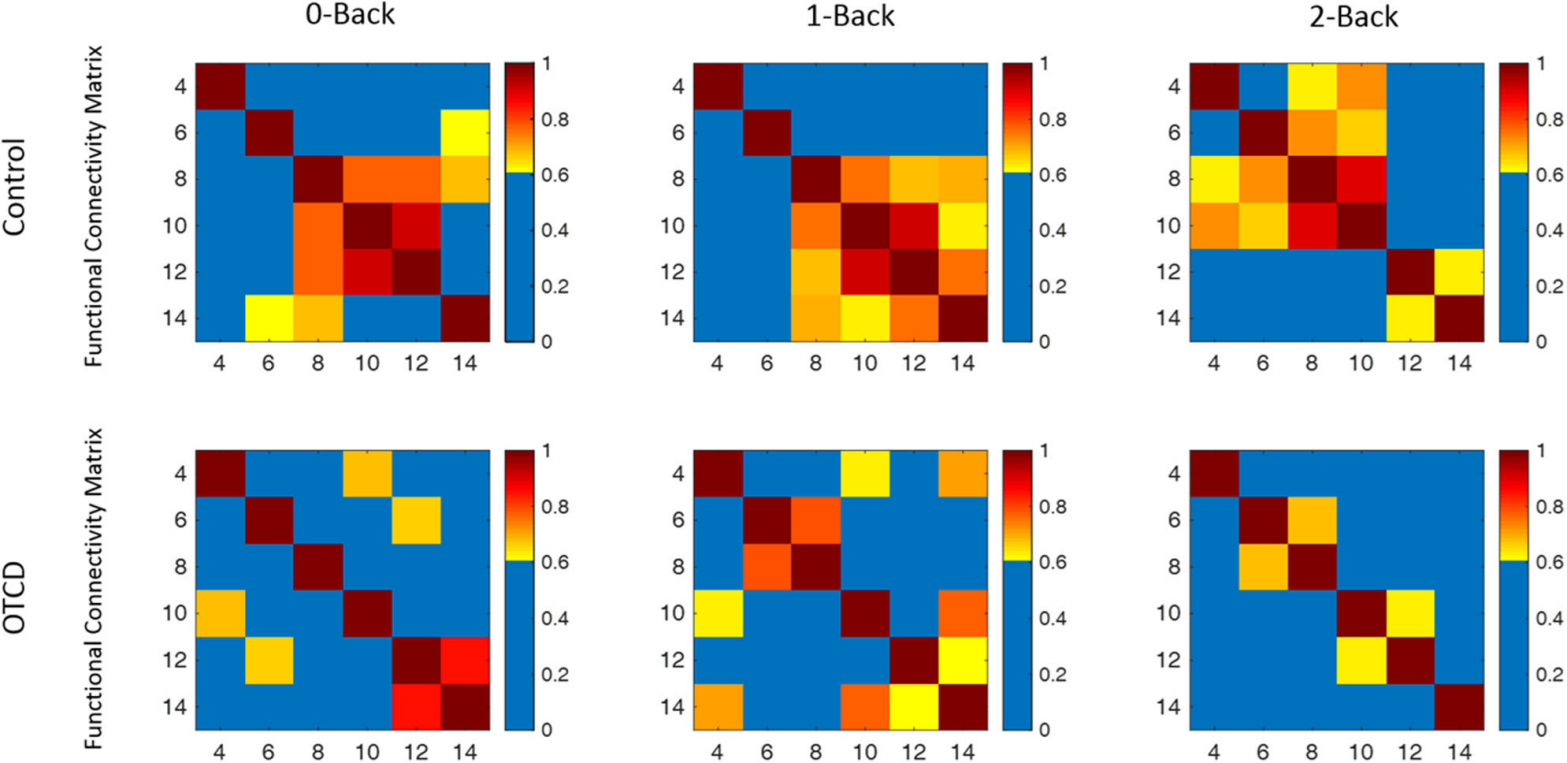
Functional connectivity map of the control and OTCD sibling during performance of 0-, 1-, and 2-back working memory tasks based on the coherence analysis between each channel total hemoglobin measurements. In the control sibling, there was an increase in functional connectivity from the 0–2-back condition, whereas in the sibling with OTCD, this pattern was not observed.

We next examined the functional connectivity in the prefrontal cortex region during performance of the working memory task. Figure 6 shows the functional connectivity map based on the correlation matrix between the channels during performance of the 0-, 1-, and 2-back WM task in the control and OTCD siblings. Compared to the OTCD sibling, the connectivity strength between two hemispheres is stronger in terms of both inter- and intra-hemispheric connectivity in the control sibling, especially during more demanding conditions, such as 1- and 2-back.

Compared to 0- and 1-back conditions, during the 2-back condition, there was a hemispheric shift from the right to the left PFC. This pattern, however, was not observed on the OTDC sibling, where there was a decrease in connectivity from the 0- to 2-back condition.

## Discussion

In this twin study, we investigated the variations in prefrontal cortex cortical hemodynamics and functional connectivity in siblings with and without OTCD, using fNIRS. We have shown the feasibility of using non-invasive optical imaging to quantify the hemodynamics of the PFC related to behavioral performance and functional connectivity in a set of twins with and without OTCD. Studies on the twins can help to control other factors that can influence the neurocognitive function, such as variation in age and environmental differences. We applied frequency analysis to examine the oscillations at different frequency range. The result showed that hemodynamic variation at low frequency, specifically in the range of below 0.03 Hz, provides the better hemodynamic contrast to differentiate the OTCD and control subjects. We further examined the functional connectivity, where we found a pattern of reduced interhemispheric connectivity in PFC regions of the sibling with OTCD as the task demand increased. In general, this feasibility study shows the applicability of fNIRS to examine the global cognitive impairments in OTCD.

The issues with cognitive shift, going from a low (0-back) to a high-demand task (1- and 2-back), that are reflected in behavioral performance (e.g., correct response) is common in subjects with OTCD (1). From a behavioral perspective, the differences in performance were similar during the less demanding 0-back task. However, with increase in the task demand, during 1- and 2-back working memory tasks, the differences in task performance and reaction time become more prominent. The *N*-back working memory task has been done in children ages 8–15 (39, 40) with average accuracy of 82–87% for control children across all tasks. In the 11-year-old control group for visual *N*-back, the hit rate of 55% for 2-back and 76% for 1-back was reported (41), and our results falls within these ranges.

The nature and mechanism of very low frequency bands has been under investigation in recent years. A study conducted in 1988 suggested the contributions from parasympathetic activity to be a dominant factor in low-frequency bands, such as 0.002–0.03 Hz. Other studies have shown the role of the sympathetic nervous system in the regulation of cerebral hemodynamics at low frequencies that are related to the function of the sympathetic neurovascular system (42, 43). The dynamic cerebral autoregulation during very low frequencies of 0.03 Hz has been shown to change during the heat stress, whereas it was unchanged during higher frequencies, such as 0.1 Hz (44). The contribution of cholinergic, neurogenic, and myogenic control, such as vasodilatation in cerebral autoregulation, have also been studied (45, 46) and have shown the neurogenic influence to be mostly responsible for control of cerebral blood flow in low-frequency bands.

In severe cases of hyperammonemia, as in patients with inherited metabolic disorders, such as UCD, regulation of cerebral blood flow is impaired (47, 48). Elevated ammonia can induce vasodilatation, which can occur in OTCD conditions (49) and can, therefore, affect cerebral autoregulation. We have used hemodynamic variation based on coefficient of variation to quantify changes in the level of total hemoglobin signal. Since using fNIRS, we are measuring the hemodynamic oscillations, and one important feature is to quantify the degree of these oscillations at low frequencies by using coefficient of variation. These low frequencies have been shown to be a correlate of underlying vasomotion and autoregulatory mechanisms (19, 50). Given that hyperammonemia influences the cerebral blood flow and autoregulatory processes (17, 47), such as vasodilation and vasoconstriction processes that can, in turn, affect these observed oscillations, quantification of these oscillations becomes important. Our results indicate higher variations in PFC hemodynamics in the OTCD sibling. The contribution of the low-frequency band below 0.03 Hz, related to cerebral autoregulation, showed higher contrast between the control and OTCD siblings compared to the band of 0.07–0.1 Hz. This conclusion is based on the fact that both VLF1 band (0.001–0.1 Hz) and that of <0.03 Hz yielded similar percentage differences compared to the frequency band of 0.07–0.1 Hz. This can further elucidate the possibility of underlying autoregulation issues, such as vasomotion and autoregulation inefficiency in the OTCD population due to elevated ammonia. Further longitudinal examination of the OTCD twin and under conditions such as pre- and post- treatment plan can be helpful in clarifying underlying mechanisms.

FC in the prefrontal cortex has been studied in several disorders, such as autism spectrum disorder (51) and obsessive-compulsive disorder (52). It has been shown that subjects with OTCD have impaired cognitive flexibility (1, 53), where it is difficult to shift from low to high demanding tasks in daily life activities. Previous studies have shown the reduced FC in ventrolateral prefrontal cortex was associated with reduced cognitive flexibility (52). Increase in functional connectivity during cognitive challenges in PFC has been shown (54). Other studies of the neural basis of working memory have shown that functional connectivity during performance of working memory in patients with schizophrenia decreased in dorsal lateral PFC (DLPFC) regions compared to healthy controls. A study by Gropman et al. (3) showed altered neural activation in OTCD during working memory performance. In line with these studies, our results have shown a decrease in PFC connectivity in the sibling with OTCD compared to the unaffected sibling. In terms of cognitive flexibility, the sibling with OTCD showed a decrease in the PFC connectivity network when shifting from low demand (0- and 1-back) to a higher demand task (2-back). Whereas, in the control sibling, our results indicate an increase in FC with the level of task difficulty. We further noticed that the FC network exhibited localized connectivity patterns in the control sibling compared to the sibling with OTCD who showed reduced and diffused FC. Results further showed an interhemispheric connectivity shift from the right PFC in the 1-back task to the left PFC during the 2-back task in the control sibling. Previous studies in subjects with OTCD have shown a decline in cognitive function with an increase in task difficulty, where the activation reaches the peak during less demanding tasks and declines as the cognitive demand increases (3). These results could be indicative of previous work (55) examining the inverted U shape, where, after a person reaches their cognitive capacity, the activation begins to decline in response to a higher demand task.

In the future, including broader cortical regions and using short distance measurements as well as increasing the population size would better clarify the differentiation in the degree of functional connectivity and hemodynamic oscillations at low frequency range. It is important to note that, although we have examined the hemodynamics of the PFC during performance of the working memory task, such measures are not necessarily representative of the working memory deficit and could reflect the global cognitive impairment due to HA. Although the general trend of lower IQ in symptomatic OTCD cohorts compared to control is known (53, 56), formal IQ testing was not performed due to lack of developmental concerns. In future studies, inclusion of additional measures, such as intelligence quotient (IQ) score, comprehensive evaluation in domains of working memory and executive function, and biochemical phenotypes, such as glutamine, arginine, and plasma ammonia at the time of imaging would further elucidate the observed cognitive results with respect to specific cognitive domains and abilities. This limited study demonstrates the applicability and feasibility of using fNIRS to verify differences in cognitive functions related to PFC in OTCD and an age-matched typically developing sibling. The applicability of this exam to test cognitive impairments (specific or global) will be evaluated in future studies.

Overall, the result of this twin study suggests the contribution of inefficient autoregulatory mechanism in underlying neurocognitive function of subjects with OTCD as well as reduced functional connectivity in PFC when faced with cognitive challenge. The results of this study imply better understanding of hemodynamic features to further distinguish the cognitive function in developmental disorders.

## Data Availability Statement

The datasets generated for this study are available on request to the corresponding author.

## Ethics Statement

The studies involving human participants were reviewed and approved by The Children's National Medical Center Institutional Review Board. Written informed consent to participate in this study was provided by the participants' legal guardian/next of kin.

## Author Contributions

AA conducted data analysis and acquisition and prepared tables and figures. AGr and AA wrote and revised the manuscript. AA, AGr, CL, CS, and AGa edited the manuscript and provided comments. AGr and AGa, and CS gave technical support and conceptual advice. AGa supervised the analysis and provided analytical insight. All authors contributed to manuscript revision, read and approved the submitted version.

## Conflict of Interest

The authors declare that the research was conducted in the absence of any commercial or financial relationships that could be construed as a potential conflict of interest.

## References

[1] Gropman AL Prust M Breeden A Fricke S VanMeter J. Urea cycle defects and hyperammonemia: effects on functional imaging. Metab Brain Dis. (2013) 28:269–75. 10.1007/s11011-012-9348-0 23149878 PMC3594356

[2] Gropman AL Sailasuta N Harris KC Abulseoud O Ross BD. Ornithine transcarbamylase deficiency with persistent abnormality in cerebral glutamate metabolism in adults. Radiology. (2009) 252:833–41. 10.1148/radiol.2523081878 19567648 PMC2734894

[3] Gropman AL Shattuck K Prust MJ Seltzer RR Breeden AL Hailu A. Altered neural activation in ornithine transcarbamylase deficiency during executive cognition: an fMRI study. Hum Brain Mapp. (2013) 34:753–61. 10.1002/hbm.21470 22110002 PMC3338900

[4] Sen K Whitehead MT Gropman AL Multimodal imaging in urea cycle-related neurological disease – what can imaging after hyperammonemia teach us? Trans Sci Rare Dis. (2020) 5:87–95. 10.3233/TRD-200048 PMC7739971 33344172

[5] Anderson AA Parsa K Geiger S Zaragoza R Kermanian R Miguel H. Exploring the role of task performance and learning style on prefrontal hemodynamics during a working memory task. PLoS ONE. (2018) 13:e0198257. 10.1371/journal.pone.0198257 29870536 PMC5988299

[6] Dashtestani H Zaragoza R Pirsiavash H Knutson KM Kermanian R Cui J. Canonical correlation analysis of brain prefrontal activity measured by functional near infra-red spectroscopy (fNIRS) during a moral judgment task. Behav Brain Res. (2019) 359:73–80. 10.1016/j.bbr.2018.10.022 30343055 PMC6482827

[7] Sherafati A Hassanpour MS Dwyer N Fishell AK Eggebrecht AT Firszt JB Optical neuroimaging of speech perception in listeners with cochlear implants. In: Biophotonics Congress: Biomedical Optics 2020 (Translational, Microscopy, OCT, OTS, BRAIN). Washington, DC: OSA (Optical Society of America) (2020). 10.1364/BRAIN.2020.BM4C.4

[8] Obrig H Neufang M Wenzel R Kohl M Steinbrink J Einhäupl K. Spontaneous low frequency oscillations of cerebral hemodynamics and metabolism in human adults. Neuroimage. (2000) 12:623–39. 10.1006/nimg.2000.0657 11112395

[9] Sassaroli A Pierro M Bergethon PR Fantini S Low-frequency spontaneous oscillations of cerebral hemodynamics investigated with near-infrared spectroscopy: a review. IEEE J SelectTopics Quant Electron. (2012) 18:1478–92. 10.1109/JSTQE.2012.2183581

[10] Chiron CRC Maziere B Zilbovicius M Laflamme L Masure MC Dulac O. Changes in regional cerebral blood flow during brain maturation in children and adolescents. J Nucl Med. (1992) 33:696–703. 1569478

[11] Udomphorn Y Armstead WM Vavilala MS. Cerebral blood flow and autoregulation after pediatric traumatic brain injury. Pediatr Neurol. (2008) 38:225–34. 10.1016/j.pediatrneurol.2007.09.012 18358399 PMC2330089

[12] Kilroy E Liu CY Yan L Kim YC Dapretto M Mendez MF. Relationships between cerebral blood flow and IQ in typically developing children and adolescents. J Cogntive Sci. (2011) 12:151–70. 10.17791/jcs.2011.12.2.151 23976891 PMC3749787

[13] Anderson AA Smith E Chernomordik V Ardeshirpour Y Chowdhry F Thurm A. Prefrontal cortex hemodynamics and age: a pilot study using functional near infrared spectroscopy in children. Front Neurosci. (2014) 8:393. 10.3389/fnins.2014.00393 25565935 PMC4266015

[14] Liu XCM Donnelly J Budohoski KP Varsos GV Nasr N Brady KM. Comparison of frequency and time domain methods of assessment of cerebral autoregulation in traumatic brain injury. J Cereb Blood Flow Metab. (2015) 35:248–56. 10.1038/jcbfm.2014.192 25407266 PMC4426741

[15] Chernomordik VAF Kenney K Wassermann E Diaz-Arrastia R Gandjbakhche A. Abnormality of low frequency cerebral hemodynamics oscillations in TBI population. Brain Res. (2016) 1639:194–9. 10.1016/j.brainres.2016.02.018 26996413 PMC9392959

[16] Anderson AA Smith E Chowdhry FA Thurm A Condy E Swineford L. Prefrontal hemodynamics in toddlers at rest: a pilot study of developmental variability. Front Neurosci. (2017) 11:300. 10.3389/fnins.2017.00300 28611578 PMC5447733

[17] Chodobski A Szmydynger-Chodobska J Skolasinska K. Effect of ammonia intoxication on cerebral blood flow, its autoregulation and responsiveness to carbon dioxide and papaverine. J Neurol Neurosurg Psychiatr. (1986) 49:302–9. 10.1136/jnnp.49.3.302 3083051 PMC1028730

[18] Bjerring PN Bjerrum EJ Larsen FS. Impaired cerebral microcirculation induced by ammonium chloride in rats is due to cortical adenosine release. J Hepatol. (2018) 68:1137–43. 10.1016/j.jhep.2018.01.034 29452205

[19] Charles D Fraser I Brady KM Rhee CJ Easley RB Kibler K. The frequency response of cerebral autoregulation. J Appl Physiol. (2013) 115:52–6. 10.1152/japplphysiol.00068.2013 23681909

[20] Andersen AV Simonsen SA Schytz HW Iversen HK. Assessing low-frequency oscillations in cerebrovascular diseases and related conditions with near-infrared spectroscopy: a plausible method for evaluating cerebral autoregulation? Neurophotonics. (2018) 5:030901. 10.1117/1.NPh.5.3.030901 30689678 PMC6156398

[21] Hull JV Dokovna LB Jacokes ZJ Torgerson CM Irimia A Van Horn JD. Resting-state functional connectivity in autism spectrum disorders: a review. Front Psychiatry. (2017) 7:205. 10.3389/fpsyt.2016.00205 28101064 PMC5209637

[22] Bos DJ Oranje B Achterberg M Vlaskamp C Ambrosino S de Reus MA. Structural and functional connectivity in children and adolescents with and without attention deficit/hyperactivity disorder. J Child Psychol Psychiatry. (2017) 58:810–8. 10.1111/jcpp.12712 28295280

[23] Svoboda AM Burns-Yocum T Sherafati A Schroeder ML Rafferty S Culver J Mapping neural correlates to language and biological motion in school-age children with autism using HD-DOT. In: Biophotonics Congress: Biomedical Optics 2020 (Translational, Microscopy, OCT, OTS, BRAIN). Washington, DC: OSA (Optical Society of America) (2020). 10.1364/TRANSLATIONAL.2020.JTh2A.33

[24] Burke B Eggebrecht A Bergonzi K Sherafati A Burns-Yocum T Fishell A Brain functional connectivity changes in acute ischemic stroke measured with bedside diffuse optical tomography. In: Journal Of Cerebral Blood Flow and Metabolism. Thousand Oaks, CA: Sage Publications INC 2455 Teller RD (2019). p. 8–9.

[25] von dem Hagen EAH Stoyanova RS Baron-Cohen S Calder AJ. Reduced functional connectivity within and between 'social' resting state networks in autism spectrum conditions. Soc Cogn Affect Neurosci. (2013) 8:694–701. 10.1093/scan/nss053 22563003 PMC3739917

[26] Mayer AR Mannell MV Ling J Gasparovic C Yeo RA Functional connectivity in mild traumatic brain injury. Hum Brain Mapp. (2011) 32:1825–35. 10.1002/hbm.21151 21259381 PMC3204375

[27] Wang Y Yin Y Sun YW Zhou Y Chen X Ding WN. Decreased prefrontal lobe interhemispheric functional connectivity in adolescents with internet gaming disorder: a primary study using resting-state fMRI. PLoS ONE. (2015) 10:e0118733. 10.1371/journal.pone.0118733 25738502 PMC4349655

[28] Zhu H Xu J Li J Peng H Cai T Li X. Decreased functional connectivity and disrupted neural network in the prefrontal cortex of affective disorders: A resting-state fNIRS study. J Affect Disord. (2017) 221:132–44. 10.1016/j.jad.2017.06.024 28645025

[29] Jiang L Geng W Chen H Zhang H Bo F Mao C-N. Decreased functional connectivity within the default-mode network in acute brainstem ischemic stroke. Eur J Radiol. (2018) 105:221–6. 10.1016/j.ejrad.2018.06.018 30017284

[30] Moerman A De Hert S Recent advances in cerebral oximetry. assessment of cerebral autoregulation with near-infrared spectroscopy: myth or reality? F1000Research. (2017) 6:1615 10.12688/f1000research.11351.1 29026526 PMC5583743

[31] Butterworth RF. Effects of hyperammonaemia on brain function. J Inherit Metab Dis. (1998) 1:6–20. 10.1023/A:1005393104494 9686341

[32] Barkovich E Robinson C Gropman A Brain biomarkers and neuroimaging to diagnose urea cycle disorders and assess prognosis. Expert Opinion Orphan Drugs. (2016) 4:1123–32. 10.1080/21678707.2016.1242407

[33] Ayaz H Shewokis PA Curtin A Izzetoglu M Izzetoglu K Onaral B. Using MazeSuite and functional near infrared spectroscopy to study learning in spatial navigation. J Visual Exp. (2011) 56:3443. 10.3791/3443 22005455 PMC3227178

[34] Scholkmann F Wolf M. General equation for the differential pathlength factor of the frontal human head depending on wavelength and age. J Biomed Optics. (2013) 18:105004. 10.1117/1.JBO.18.10.105004 24121731

[35] Sato H Yahata N Funane T Takizawa R Katura T Atsumori H. A NIRS–fMRI investigation of prefrontal cortex activity during a working memory task. Neuroimage. (2013) 83:158–73. 10.1016/j.neuroimage.2013.06.043 23792984

[36] Boashash B Estimating and interpreting the instantaneous frequency of a signal. I fundamentals Proc IEEE. (1992) 80:520–38. 10.1109/5.135376

[37] Bu L Li J Li F Liu H Li Z. Wavelet coherence analysis of cerebral oxygenation signals measured by near-infrared spectroscopy in sailors: an exploratory, experimental study. BMJ Open. (2016) 6:e013357. 10.1136/bmjopen-2016-013357 27810980 PMC5128848

[38] Xu G Zhang M Wang Y Liu Z Huo C Li Z. Functional connectivity analysis of distracted drivers based on the wavelet phase coherence of functional near-infrared spectroscopy signals. PLoS ONE. (2017) 12:e0188329. 10.1371/journal.pone.0188329 29176895 PMC5703451

[39] Zhang K Ma J Lei D Wang M Zhang J Du X. Task positive and default mode networks during a working memory in children with primary monosymptomatic nocturnal enuresis and healthy controls. Pediatr Res. (2015) 78:422–9. 10.1038/pr.2015.120 26086645

[40] Barnes JJ Nobre AC Woolrich MW Baker K Astle DE. Training working memory in childhood enhances coupling between frontoparietal control network and task-related regions. J Neurosci. (2016) 36:9001. 10.1523/jneurosci.0101-16.2016 27559180 PMC4995310

[41] Pelegrina S Lechuga MT García-Madruga JA Elosúa MR Macizo P Carreiras M. Normative data on the n-back task for children and young adolescents. Front Psychol. (2015) 6:1544. 10.3389/fpsyg.2015.01544 26500594 PMC4597481

[42] Saleem S Teal PD Kleijn WB Ainslie PN Tzeng Y-C. Identification of human sympathetic neurovascular control using multivariate wavelet decomposition analysis. Am J Physiol-Heart Circul Physiol. (2016) 311:H837–48. 10.1152/ajpheart.00254.2016 27317632

[43] Saleem S Tzeng Y-C Kleijn WB Teal PD. Detection of impaired sympathetic cerebrovascular control using functional biomarkers based on principal dynamic mode analysis. Front Physiol. (2017) 7:685. 10.3389/fphys.2016.00685 28119628 PMC5220091

[44] Brothers RM Zhang R Wingo JE Hubing KA Crandall CG. Effects of heat stress on dynamic cerebral autoregulation during large fluctuations in arterial blood pressure. J Appl Physiol. (2009) 107:1722–9. 10.1152/japplphysiol.00475.2009 19797691 PMC2793195

[45] Hamner JW Tan CO Tzeng Y-C Taylor JA. Cholinergic control of the cerebral vasculature in humans. J Physiol. (2012) 590:6343–52. 10.1113/jphysiol.2012.245100 23070700 PMC3533196

[46] Hamner JW Tan CO Relative contributions of sympathetic, cholinergic, and myogenic mechanisms to cerebral autoregulation. Stroke. (2014) 45:1771–7. 10.1161/STROKEAHA.114.005293 24723314 PMC4102642

[47] Larsen FS. Cerebral blood flow in hyperammonemia: heterogeneity and starling forces in capillaries. Metab Brain Dis. (2002) 17:229–35. 10.1023/A:1021941414605 12602500

[48] Vaquero J Chung C Blei AT. Cerebral blood flow in acute liver failure: a finding in search of a mechanism. Metab Brain Dis. (2004) 19:177–94. 10.1023/B:MEBR.0000043968.04313.e7 15554414

[49] Andersson K-E Brandt L Hindfelt B Ljunggren B. Cerebrovascular effects of ammonia *in vitro*. Acta Physiol Scand. (1981) 113:349–53. 10.1111/j.1748-1716.1981.tb06906.x 7345901

[50] Bassan H Gauvreau K Newburger JW Tsuji M Limperopoulos C Soul JS. Identification of pressure passive cerebral perfusion and its mediators after infant cardiac surgery. Pediatr Res. (2005) 57:35–41. 10.1203/01.PDR.0000147576.84092.F9 15531739

[51] Negrón-Oyarzo I Aboitiz F Fuentealba P. Impaired functional connectivity in the prefrontal cortex: a mechanism for chronic stress-induced neuropsychiatric disorders. Neural Plast. (2016) 2016:7539065–7539065. 10.1155/2016/7539065 26904302 PMC4745936

[52] Vaghi MM Vértes PE Kitzbichler MG Apergis-Schoute AM van der Flier FE Fineberg NA. Specific frontostriatal circuits for impaired cognitive flexibility and goal-directed planning in obsessive-compulsive disorder: evidence from resting-state functional connectivity. Biol Psychiatry. (2017) 81:708–17. 10.1016/j.biopsych.2016.08.009 27769568 PMC6020061

[53] Sprouse C King J Helman G Pacheco-Colón I Shattuck K Breeden A. Investigating neurological deficits in carriers and affected patients with ornithine transcarbamylase deficiency. Mol Genet Metab. (2014) 113:136–41. 10.1016/j.ymgme.2014.05.007 24881970 PMC4458385

[54] Racz FS Mukli P Nagy Z Eke A. Increased prefrontal cortex connectivity during cognitive challenge assessed by fNIRS imaging. Biomed Opt Express. (2017) 8:3842–55. 10.1364/boe.8.003842 28856054 PMC5560845

[55] Callicott JH Mattay VS Verchinski BA Marenco S Egan MF Weinberger DR Complexity of prefrontal cortical dysfunction in schizophrenia: more than up or down. Am J Psychiatry. (2003) 160:2209–15. 10.1176/appi.ajp.160.12.2209 14638592

[56] Krivitzky L Babikian T Lee H-S Thomas NH Burk-Paull KL Batshaw ML. Intellectual, adaptive, and behavioral functioning in children with urea cycle disorders. Pediatr Res. (2009) 66:96–101. 10.1203/PDR.0b013e3181a27a16 19287347 PMC2746951

